# Supplementary material for: Clinical efficacy and safety of organ-sparing cystectomy: a systematic review and meta-analysis
Source: PeerJ. 2024 Nov 27;12:e18427. doi: 10.7717/peerj.18427 (PMC11639212; doi:10.7717/peerj.18427)
Supplement: Supplemental Information 3 [file peerj-12-18427-s003.docx]

**Identification of studies via databases and registers**

Records removed *before screening*:

Duplicate records removed (n =102 )

Records marked as ineligible by automation tools (n =206 )

Records removed for other reasons (n =0 )

Records identified from:

Pubmed (n=249)

Embase (n=542)

Web of science (n=484)

Citation searching (n=5)

**Identification**

Records screened

(n = 972)

Records excluded

(n = 930)

Reports sought for retrieval

(n = 42)

Reports not retrieved

(n =5 )

**Screening**

Reports assessed for eligibility

(n =37 )

Reports excluded:

Studies without comparison of ORC and SRC techniques (n =9 )

Studies without sufficient data (n = 9)

Studies included in review

(n =19 )

**Included**

*Consider, if feasible to do so, reporting the number of records identified from each database or register searched (rather than the total number across all databases/registers).

**If automation tools were used, indicate how many records were excluded by a human and how many were excluded by automation tools.

*From:*  Page MJ, McKenzie JE, Bossuyt PM, Boutron I, Hoffmann TC, Mulrow CD, et al. The PRISMA 2020 statement: an updated guideline for reporting systematic reviews. BMJ 2021;372:n71. doi: 10.1136/bmj.n71

For more information, visit: <http://www.prisma-statement.org/>
