# Supplementary material for: Clinical efficacy and safety of organ-sparing cystectomy: a systematic review and meta-analysis
Source: PeerJ. 2024 Nov 27;12:e18427. doi: 10.7717/peerj.18427 (PMC11639212; doi:10.7717/peerj.18427)
Supplement: Table S1 [file peerj-12-18427-s006.docx]

| \| **Table S1 Detailed search strategy in three databases** \| \| --- \| | |
| --- | --- | --- |
| Database | Search strategy |
| Pubmed | (((((((((((((((((((((((((((((Urinary Bladder Neoplasms[MeSH Terms]) OR (Neoplasm, Urinary Bladder[Title/Abstract])) OR (Urinary Bladder Neoplasm[Title/Abstract])) OR (Bladder Tumors[Title/Abstract])) OR (Bladder Tumor[Title/Abstract])) OR (Tumor, Bladder[Title/Abstract])) OR (Tumors, Bladder[Title/Abstract])) OR (Neoplasms, Bladder[Title/Abstract])) OR (Bladder Neoplasms[Title/Abstract])) OR (Bladder Neoplasm[Title/Abstract])) OR (Neoplasm, Bladder[Title/Abstract])) OR (Urinary Bladder Cancer[Title/Abstract])) OR (Cancer, Urinary Bladder[Title/Abstract])) OR (Malignant Tumor of Urinary Bladder[Title/Abstract])) OR (Cancer of the Bladder[Title/Abstract])) OR (Bladder Cancer[Title/Abstract])) OR (Bladder Cancers[Title/Abstract])) OR (Cancer, Bladder[Title/Abstract])) OR (Cancer of Bladder[Title/Abstract])) OR (Cystectomy[MeSH Terms])) OR (Cystectomies[Title/Abstract])) OR (Partial Cystectomy[Title/Abstract])) OR (Cystectomy, Partial[Title/Abstract])) OR (Partial Cystectomies[Title/Abstract])) OR (Radical Cystectomy[Title/Abstract])) OR (Cystectomy, Radical[Title/Abstract])) OR (Radical Cystectomies[Title/Abstract])) AND ((((((((((Prostate[Title/Abstract]) OR (Capsule[Title/Abstract])) OR (seminal vesicles[Title/Abstract])) OR (neurovascular bundle[Title/Abstract])) OR (NVB[Title/Abstract])) OR (Nerve[Title/Abstract])) OR (Uterus[Title/Abstract])) OR (fallopian tubes[Title/Abstract])) OR (Ovaries[Title/Abstract])) OR (Vagina[Title/Abstract]))) AND (((((Sparing[Title/Abstract]) OR (Protect[Title/Abstract])) OR (Reserve[Title/Abstract])) OR (Preserve[Title/Abstract])) OR (spare[Title/Abstract]))) NOT ((((Hysterectomy[MeSH Terms]) OR (prostatectomy[MeSH Terms])) OR (Prostate Cancer[MeSH Terms])) OR (Uterus Cancer[MeSH Terms])) |
|  | #1 Urinary Bladder Neoplasms(Topic) OR Neoplasm, Urinary Bladder(Abstract) OR Urinary Bladder Neoplasm(Abstract) OR Bladder Tumors(Abstract) OR Bladder Tumor(Abstract) OR Tumor, Bladder(Abstract) OR Tumors, Bladder(Abstract) OR Neoplasms, Bladder(Abstract) OR Bladder Neoplasms(Abstract) OR Bladder Neoplasm(Abstract) OR Neoplasm, Bladder(Abstract) OR Urinary Bladder Cancer(Abstract) OR Cancer, Urinary Bladder(Abstract) OR Malignant Tumor of Urinary Bladder(Abstract) OR Cancer of the Bladder(Abstract) OR Bladder Cancer(Abstract) OR Bladder Cancers(Abstract) OR Cancer, Bladder(Abstract) OR Cancer of Bladder (Abstract) OR Cystectomy(Topic) OR Cystectomies(Abstract) OR Partial Cystectomy(Abstract) OR Cystectomy, Partial(Abstract) OR Partial Cystectomies(Abstract) OR Radical Cystectomy(Abstract) OR Cystectomy, Radical(Abstract) OR Radical Cystectomies(Abstract) |
| Web of sci | #2 Prostate(Abstract) OR Capsule(Abstract) OR seminal vesicles(Abstract) OR neurovascular bundle(Abstract) OR NVB(Abstract) OR Nerve(Abstract) OR Uterus(Abstract) OR fallopian tubes(Abstract) OR Ovaries(Abstract) OR Vagina(Abstract) |
|  | #3 Sparing(Abstract) OR Protect(Abstract) OR Reserve(Abstract) OR Preserve(Abstract) OR Spare(Abstract) |
|  | #4 Hysterectomy(Topic) OR Prostatectomy(Topic) OR Prostate Cancer(Topic) OR Uterus Cancer(Topic) |
|  | #5 #1 AND #2 AND #3 |
|  | #6 #5 NOT #4 |
| Embase | (('urinary bladder neoplasms'/exp OR 'neoplasm,urinary bladder':ti,ab OR 'bladder tumors':ti,ab OR 'bladder tumor':ti,ab OR 'tumor,bladder':ti,ab OR 'tumors, bladder':ti,ab OR 'neoplasms, bladder':ti,ab OR 'bladder neoplasms':ti,ab OR 'bladder neoplasm':ti,ab OR 'neoplasm, bladder':ti,ab OR 'urinary bladder cancer':ti,ab OR 'cancer, urinary bladder':ti,ab OR 'malignant tumor of urinary bladder':ti,ab OR 'cancer of the bladder':ti,ab OR 'bladder cancer':ti,ab OR 'bladder cancers':ti,ab OR 'cancer, bladder':ti,ab OR 'cancer of bladder':ti,ab OR 'cystectomy'/exp OR 'cystectomies':ti,ab OR 'partial cystectomy':ti,ab OR 'cystectomy, partial':ti,ab OR 'partial cystectomies':ti,ab OR 'cystectomy, radical':ti,ab OR 'radical cystectomies':ti,ab) AND ('prostate':ti,ab OR 'capsule':ti,ab OR 'seminal vesicles':ti,ab OR 'nvb':ti,ab OR 'neurovascular bundle':ti,ab OR 'nerve':ti,ab OR 'uterus':ti,ab OR 'fallopian tubes':ti,ab OR 'ovaries':ti,ab OR 'vagina':ti,ab) AND ('sparing':ti,ab OR 'protect':ti,ab OR 'reserve':ti,ab OR 'preserve':ti,ab OR 'spare':ti,ab)) NOT ('hysterectomy'/exp OR 'prostatectomy'/exp OR 'prostate cancer'/exp OR 'uterus cancer'/exp) |
