# Supplementary material for: Clinical efficacy and safety of organ-sparing cystectomy: a systematic review and meta-analysis
Source: PeerJ. 2024 Nov 27;12:e18427. doi: 10.7717/peerj.18427 (PMC11639212; doi:10.7717/peerj.18427)
Supplement: Table S2 [file peerj-12-18427-s007.docx]

| **TABLE S2 Quality score of included studies based on the Newcastle–Ottawa Scale** | | | | | | | | | | | | | | | | |  |
| --- | --- | --- | --- | --- | --- | --- | --- | --- | --- | --- | --- | --- | --- | --- | --- | --- | --- |
|  | |  | Selection | |  | Comparability | | | | |  | Exposure | |  | Total stars | |  |
| References | | REC | SNEC | | AE | DO | | SC | AF | | AO | FU | | AFU |  | |  |
| Jae et al.2022 | | 1 | 1 | | 1 | 1 | | 1 |  | | 1 | 1 | | 1 | 8 | |  |
| Po et al.2017 | | 1 | 1 | | 1 | 1 | | 1 |  | | 1 | 1 | | 1 | 8 | |  |
| Vilaseca et al.2013 | | 1 | 1 | | 1 | 1 | | 1 |  | | 1 |  | | 1 | 7 | |  |
| Se et al.2018 | | 1 | 1 | | 1 | 1 | |  |  | | 1 | 1 | | 1 | 7 | |  |
| Kyung et al.2005 | | 1 | 1 | | 1 | 1 | | 1 |  | | 1 | 1 | | 1 | 8 | |  |
| Marc et al.2018 | | 1 | 1 | | 1 | 1 | | 1 | 1 | | 1 | 1 | |  | 8 | |  |
| Qiang et al.2022 | | 1 | 1 | | 1 | 1 | | 1 |  | | 1 | 1 | | 1 | 8 | |  |
| Abbas et al.2012 | | 1 |  | | 1 | 1 | | 1 |  | | 1 | 1 | |  | 6 | |  |
| Katharina et al.2021 | | 1 | 1 | | 1 | 1 | | 1 |  | | 1 |  | | 1 | 7 | |  |
| Remco et al.2009 | | 1 | 1 | | 1 | 1 | | 1 |  | | 1 | 1 | | 1 | 8 | |  |
| Ihab et al.2008 | | 1 | 1 | | 1 | 1 | | 1 |  | | 1 | 1 | | 1 | 8 | |  |
| Sunil et al.2022 | | 1 | 1 | | 1 | 1 | | 1 | 1 | | 1 | 1 | | 1 | 9 | |  |
| William et al.1997 | | 1 | 1 | | 1 | 1 | |  |  | | 1 | 1 | | 1 | 7 | |  |
| Song et al.2018 | | 1 | 1 | | 1 | 1 | | 1 | 1 | | 1 | 1 | | 1 | 9 | |  |
| Magdy et al.2006 | | 1 | 1 | | 1 | 1 | |  |  | | 1 | 1 | |  | 6 | |  |
| Thomas et al.2004 | | 1 | 1 | | 1 | 1 | |  |  | | 1 | 1 | | 1 | 7 | |  |
| Wang et al.2008 | | 1 | 1 | | 1 | 1 | | 1 |  | | 1 |  | | 1 | 7 | |  |
| Haiwen et al.2019 | | 1 | 1 | | 1 | 1 | | 1 |  | | 1 | 1 | | 1 | 8 | |  |
| AE, ascertainment of exposure; AF, study controls for other important factors; AFU, adequacy of followup of cohort ( ≥ 80%); AO, assessment of outcome;DO, demonstration that outcome of interest was not present at start of study; FU, follow-up long enough for outcomes to occur;REC, representativeness of the cohort; SC, study controls most important factors; SNEC, selection of the none posed cohort. | | | | | | | | | | | | | | | | |  |
|  | | | | | | | | | | | | | | | | |  |
|  | | | | | | | | | | | | | | | | |  |
| **Risk of bias for RCTs as measured by the Cochrane Risk of Bias 2.0 tool** | | | | | | | | | | | | | | | | | |
|  | Randomization process | | | Deviations from intended interventions | | | Missing  outcome data | | | Measurement of  the outcome | | | Selection of the  reported result | | |  | |
| References |  |  |  |  |  |  |  |  |  |  |  |  |  |  |  | Overall | |
| Ahmed et al.2019 | Low risk | | | Low risk | | | Low risk | | | Low risk | | | Low risk | | | Low risk | |
