# Supplementary material for: Clinical efficacy and safety of organ-sparing cystectomy: a systematic review and meta-analysis
Source: PeerJ. 2024 Nov 27;12:e18427. doi: 10.7717/peerj.18427 (PMC11639212; doi:10.7717/peerj.18427)
Supplement: Table S3 [file peerj-12-18427-s008.docx]

| **TABLE S3 Clinical and pathological characteristics of included studies** | | | | | | | | | | | | |
| --- | --- | --- | --- | --- | --- | --- | --- | --- | --- | --- | --- | --- |
| **Authors** | **CT** | | | **PT** | | | **PN** | | | **Age(years)** | | |
|  | **OSC** | **SRC** | **P_1_** | **OSC** | **SRC** | **P_2_** | **OSC** | **SRC** | **P_3_** | **OSC** | **SRC** | **P_4_** |
| **Jae et al. 2022**^19^ | ≤ T2:37  > T2:3 | ≤ T2:34  > T2:12 | 0.33 | ≤ T2:28  > T2:12 | ≤ T2:26  > T2:20 | 0.21 | N0:33  N+:7 | N0:42  N+:4 | 0.19 | 69(Iqr33-84) | 69(Iqr44-89) | 0.91 |
| **Po et al. 2017**^20^ | - | - |  | < T2:5  ≥ T2:9 | < T2:6  ≥ T2:5 | 0.437  0.351 | - | - |  | 57.5 ± 13.93 | 61.55 ±15.03 | 0.695 |
| **Ahmed et al. 2019**^21^ | ≤ T2:45  > T2:0 | ≤ T2:51  > T2:0 | 0.4 | - | - |  | - | - |  | 62.2 ± 5.8 | 63.7 ± 6.8 | 0.07 |
| **Vilaseca et al. 2013**^22^ | < T2:1  ≥ T2:10 | < T2:2  ≥ T2:31 | 0.73 | < T2:24  ≥ T2:20 | | - | N0:37  N+:7 | | - | 49.4±9.1 | 56.1 ± 5.9 | 0.007* |
| **Se et al. 2018**^23^ | ≤ T2:15  > T2:0 | ≤ T2:20  > T2:3 | 0.073 | ≤ T2:10  > T2:5 | ≤ T2:11  > T2:12 | 0.065 | N0:11  N+:4 | N0:17  N+:6 | 0.999 | 58.7+-4.96 | 61.9+-5.9 | 0.209 |
| **Kyung et al. 2005**^24^ | ≤ T2:17  > T2:0 | ≤ T2:18  > T2:0 | - | - | - |  | - | - |  | 54.4 (range44-66) | 59.4 (range46-68) | - |
| **Marc et al. 2018**^25^ | - | - |  | ≤ T2:125  > T2:25 | ≤ T2:17  > T2:7 | 0.005* | N0:128  N+:22 | N0:22  N+:2 | 0.2 | 61.5(Iqr57-70) | 65(Iqr63-69) | 0.5 |
| **Qiang et al. 2022**^26^ | - | - |  | ≤ T2:11  > T2:0 | ≤ T2:21  > T2:1 | - | N0:11  N+:0 | N0:22  N+:0 | - | 53 (Iqr49‒57) | 58(Iqr50‒63) | 0.089 |
| **Abbas et al. 2012**^27^ | ≤ T2:15  - | ≤ T2:21  - | 0.31 | ≤ T2:17  > T2:6 | ≤ T2:18  > T2:9 | 0.51 | - | - |  | 59±14.0 | 61±12.0 | 0.59 |
| **Katharina et al. 2021**^28^ | - | - |  | ≤ T2:36  > T2:12 | ≤ T2:47  > T2:21 | 0.489 | N0:40  N+:8 | N0:53  N+:15 | 0.458 | 59.5(Iqr54-65) | 62(57-68) | 0.818 |
| **Remco et al. 2009**^29^ | ≤ T2:53  > T2:10 | ≤ T2:48  > T2:15 | - | ≤ T2:47  > T2:16 | ≤ T2:41  > T2:22 | - | N0:45  N+:18 | N0:51  N+:12 | - | 55 | 61 | <0.002* |
| **Ihab et al. 2008**^30^ | - | - |  | ≤ T2:10  > T2:11 | ≤ T2:11  > T2:13 | 0.419 | N0:19  N+:2 | N0:20  N+:4 | 0.422 | 48.4 (range31-54 ) | 52.5 ( range45-64) | 0.14 |
| **Sunil et al. 2022**^31^ | ≤T2:171> T2:4 | ≤ T2:84  > T2:5 | 0.81 | ≤ T2:124  > T2:61 | ≤ T2:42  > T2:59 | <0.05* | N0:146  N+:31 | N0:69  N+:29 | 0.03* | 68  (range37-88) | 67 (range40-83) | 0.16 |
| **William et al. 1997**^32^ | ≤ T2:59  >T2:106 | - |  | - | - |  | - | - |  | 63  (range40-79) | 68  (range39-83) | - |
| **Song et al. 2018**^33^ | - | - |  | ≤ T2:36  > T2:9 | ≤ T2:36  > T2:9 | 1.0 | N0:39  N+:6 | N0:42  N+:3 | 0.591 | 77.7±2.6 | 78.8±4.2 | 0.132 |
| **Magdy et al. 2006**^34^ | - | - |  | - | - |  | - | - |  | - | - |  |
| **Thomas et al. 2004**^35^ | - | - |  | ≤ T2:160  > T2:171 | | - | N0:252  N+:79 | | - | 65.2 ((Iqr58.2–70.9) | | - |
| **Wang et al. 2008**^36^ | - | - |  | ≤ T2:14  > T2:13 | ≤ T2:5  > T2:4 | - | - | - |  | 47 | 46 | - |
| **Haiwen et al. 2019**^37^ | ≤ T2:44  > T2:19 | ≤ T2:28  > T2:21 | 0.017* | ≤ T2:40  > T2:23 | ≤ T2:29  > T2:20 | 0.613 | N0:60  N+:3 | N0:37  N+:12 | 0.002* | 63.7±12.2 | 67.7±9.2 | 0.855 |
| Abbreviations: cT, clinical T stage; SRC,Standard Radical Cystectomy ; OSC, Organ Sparing Cystectomy; pN, pathological N stage; pT, pathological T stage. | | | | | | | | | | | | |
