# Supplementary material for: Clinical efficacy and safety of organ-sparing cystectomy: a systematic review and meta-analysis
Source: PeerJ. 2024 Nov 27;12:e18427. doi: 10.7717/peerj.18427 (PMC11639212; doi:10.7717/peerj.18427)
Supplement: Table S4 [file peerj-12-18427-s009.docx]

| **TABLE S4 Quality of evidence for each outcome as assessed by the GRADE system** | | | | | | | |
| --- | --- | --- | --- | --- | --- | --- | --- |
| **Outcome** | **No. studies** | **Risk**  **of bias** |  |  |  |  | **Overall** |
|  | **(no.patients)** |  | **Imprecision** | **Inconsistency** | **Indirectness** | **Publication bias** | **GRADE rating** |
| **operating time** | 8（556） | moderate | low | moderate | low | low | low |
| **length of stay** | 5（308） | moderate | low | low | low | low | moderate |
| **EBL** | 6（598） | moderate | moderate | high | low | low | very low |
| **complications** | 5（389） | moderate | low | moderate | low | low | low |
| **recurrence rate** | 9（847） | moderate | low | low | low | low | moderate |
| **positive surgical margin rate** | 6（762） | moderate | low | low | low | low | moderate |
| **overall survival** | 6（274） | moderate | low | low | low | low | moderate |
| **CSS** | 5（483） | moderate | low | low | low | low | moderate |
| **daily continence（6 month）** | 8（932） | moderate | low | low | low | low | moderate |
| **nighttime continence（6 month）** | 8（933） | moderate | low | moderate | low | low | low |
| **daily continence（12 month）** | 7（890） | moderate | low | low | low | low | moderate |
| **nighttime continence（12 month）** | 7（890） | moderate | low | moderate | low | low | low |
| **CIC rate** | 5（389） | moderate | low | high | low | low | very low |
| **erectile function（≤1year）** | 4（498） | moderate | moderate | high | low | low | very low |
| **erectile function（＞1year）** | 8（872） | moderate | low | moderate | low | low | low |
| GRADE = Grading of Recommendations, Assessment, Development and Evaluations;EBL,estimated blood loss;CSS,cancer specific survival;CIC rate,Clean Intermittent Catheterization(CIC) rate. | | | | | | | |
